# Characterization of Constitutive Promoters for *piggyBac* Transposon-Mediated Stable Transgene Expression in Mesenchymal Stem Cells (MSCs)

**DOI:** 10.1371/journal.pone.0094397

**Published:** 2014-04-08

**Authors:** Sheng Wen, Hongmei Zhang, Yasha Li, Ning Wang, Wenwen Zhang, Ke Yang, Ningning Wu, Xian Chen, Fang Deng, Zhan Liao, Junhui Zhang, Qian Zhang, Zhengjian Yan, Wei Liu, Zhonglin Zhang, Jixing Ye, Youlin Deng, Guolin Zhou, Hue H. Luu, Rex C. Haydon, Lewis L. Shi, Tong-Chuan He, Guanghui Wei

**Affiliations:** 1 Stem Cell Biology and Therapy Laboratory of Ministry of Education Key Laboratory for Pediatrics, Chongqing Stem Cell Therapy and Engineering Center, and Department of Urology, The Children's Hospital of Chongqing Medical University, Chongqing, China; 2 Chongqing Key Laboratory for Oral Diseases and Biomedical Sciences, and the Affiliated Hospital of Stomatology, Chongqing Medical University, Chongqing, China; 3 Molecular Oncology Laboratory, Department of Orthopaedic Surgery and Rehabilitation Medicine, The University of Chicago Medical Center, Chicago, Illinois, United States of America; 4 Ministry of Education Key Laboratory of Diagnostic Medicine and School of Clinical Diagnostic Medicine, and the Affiliated Hospitals, Chongqing Medical University, Chongqing, China; 5 Departments of Cell Biology and Oncology of the Affiliated Southwest Hospital, the Third Military Medical University, Chongqing, China; 6 Department of Orthopaedic Surgery, the Affiliated Xiang-Ya Hospital of Central South University, Changsha, China; 7 Department of Surgery, the Affiliated Zhongnan Hospital of Wuhan University, Wuhan, China; 8 School of Bioengineering, Chongqing University, Chongqing, China; Rush University Medical Center, United States of America

## Abstract

Multipotent mesenchymal stem cells (MSCs) can undergo self-renewal and give rise to multi-lineages under given differentiation cues. It is frequently desirable to achieve a stable and high level of transgene expression in MSCs in order to elucidate possible molecular mechanisms through which MSC self-renewal and lineage commitment are regulated. Retroviral or lentiviral vector-mediated gene expression in MSCs usually decreases over time. Here, we choose to use the *piggyBac* transposon system and conduct a systematic comparison of six commonly-used constitutive promoters for their abilities to drive RFP or firefly luciferase expression in somatic HEK-293 cells and MSC iMEF cells. The analyzed promoters include three viral promoters (CMV, CMV-IVS, and SV40), one housekeeping gene promoter (UbC), and two composite promoters of viral and housekeeping gene promoters (hEFH and CAG-hEFH). CMV-derived promoters are shown to drive the highest transgene expression in HEK-293 cells, which is however significantly reduced in MSCs. Conversely, the composite promoter hEFH exhibits the highest transgene expression in MSCs whereas its promoter activity is modest in HEK-293 cells. The reduced transgene expression driven by CMV promoters in MSCs may be at least in part caused by DNA methylation, or to a lesser extent histone deacetlyation. However, the hEFH promoter is not significantly affected by these epigenetic modifications. Taken together, our results demonstrate that the hEFH composite promoter may be an ideal promoter to drive long-term and high level transgene expression using the *piggyBac* transposon vector in progenitor cells such as MSCs.

## Introduction

Mesenchymal stem cells (MSCs) are multipotent progenitors and can undergo self-renewal and differentiate into multi-lineages, including osteogenic, chondrogenic, and adipogenic lineages [1]–[6]. While MSCs have been isolated from numerous tissues, one of the major sources in adults is bone marrow stromal cells. Several major signaling pathways, such as bone morphorgenetic proteins (BMPs) and Wnt, participate in regulating the lineage-specific commitment and differentiation [3], [6]–[10] although the detailed molecular mechanisms governing MSC self-renewal and differentiation remain to be thoroughly understood.

Genetic manipulations involving in stable transgene expression are usually desired to dissect the regulatory circuitry of MSC biology. However, it has been well documented that stable transgene expression in stem cells or progenitor cells is silenced or significantly reduced over time [11], [12]. For example, in embryonic stem cells (ESCs) the control of the gene expression program that establishes and maintains ESC state is dependent on a small number of master transcription factors as most of the chromatin is in a repression state [13]–[17]. Conventional stable transgene expression approaches usually involve in the employment of retroviral or lentiviral vector to generate stable integration in stem cells. However, transgene expression is often low or reduced over time [11], [12]. This phenomenon may be caused by either single or low copy numbers of transgenes integrated into host genome, and/or epigenetic modifications of the constitutive promoters (either viral promoters or non-viral housekeeping gene promoters) [13]–[15], [18].

In this study, in order to achieve high level and stable transgene expression in progenitor cells such as MSCs, we choose to use the recently developed *piggyBac* transposon system, which can effectively integrate multiple copies of the transgene into relatively AT-rich regions of host chromosomes [19], [20]. We systematically analyzed six types of constitutive promoters for their abilities to drive the expression of reporter genes RFP and firefly luciferase in somatic HEK-293 cells and MSC-like iMEF cells. We have found that cytomegalovirus (CMV) derived promoters drive the highest transgene expression in HEK-293 cells, which is however significantly reduced in MSCs. Conversely, the human elongation factor-1α/HIV enhancer composite promoter (hEFH) exhibits the highest transgene expression in MSCs whereas its promoter activity is modest in HEK-293 cells. Further analysis indicates that the reduced transgene expression driven by CMV promoters in MSCs may be at least in part caused by DNA methylation, or to a lesser extent histone deacetlyation. The hEFH promoter, on the other hand, is not significantly affected by these epigenetic modifications. Thus, our results demonstrate that the hEFH composite promoter may be an ideal promoter to drive long-term transgene expression using the *piggyBac* transposon vectors in stem cells such as MSCs.

## Materials and Methods

### Cell culture and chemicals

HEK-293 cells were from ATCC (Manassas, VA). The iMEFs were immortalized mouse embryonic fibroblasts as previously described [21]. The cell lines were maintained in the conditions as described [21]–[26]. Trichostatin A (TSA) and 5-azacytodine were purchased from Sigma-Aldrich (St Louis, MO). Unless indicated otherwise, all chemicals were purchased from Sigma-Aldrich or Fisher Scientific (Pittsburgh, PA).

### Construction of *piggyBac* vectors expressing RFP or firefly luciferase driven by different promoters

A homemade *piggyBac* vector pMPB, which contains the *piggyBac* terminal repeats (PB-TRs), core insulators (CIs) and blasticidin B selection maker (BSD), was used as the base vector. DNA fragments containing CMV (from pEGFP-N1), CMV-IVS (from pcDNA6/TR), hEFH (from pSOS vector) [27], SV40 (from pcDNA6/TR), and UbC (from pNEBR-R1) were PCR amplified and subcloned into the pMPB vector, in front of the coding region of monomeric RFP (mRFP, or RFP) or firefly luciferase (FLuc). The CAG-hEFH composite promoter was constructed by subcloning the CAG promoter (CAG from pCX-EGFP vector) [28], [29] in front of the hEFH promoter. The constructed RFP expression vectors include pMPB-CMV-RFP, pMPB-CMV-IVS-RFP, pMPB-SV40-RFP, pMPB-UbC-RFP, pMPB-hEFH-RFP, and pMPB-CAG-hEFH-RFP. Similarly, the constructed firefly luciferase expression vectors include pMPB-CMV-FLuc, pMPB-CMV-IVS-FLuc, pMPB-SV40-FLuc, pMPB-UbC-FLuc, pMPB-hEFH-FLuc, and pMPB-CAG-hEFH-FLuc. All PCR amplified fragments were verified by DNA sequencing. Detailed information regarding vector constructions is available upon request.

### Establishment of HEK-293 and iMEF stable lines expressing RFP or firefly luciferase

To generate HEK-293 and iMEF stable cells, the above pMPB based vectors were co-transfected with a *piggyBac* transposase expression vector (System Biosciences, Mountain View, CA) into HEK-293 cells or iMEF cells with Lipofectamine (Invitrogen). Stably cells were selected in the presence of Blasticidin S. The stable pools for each promoter/cell line were scaled up, and the frozen cell stocks were kept in LN_2_ tanks.

### Genomic DNA isolation and PCR analysis

Genomic DNA was isolated from the HEK-293 and iMEFs stable lines using alkaline lysis protocol. Briefly, one confluent well of 6-well plates for each stable cell line was collected and lyzed in freshly prepared 0.2M NaOH/1 mM EDTA at 85°C for 20 min. The cell lysate was extracted with PC-8 (phenol:chloroform, pH 8.0), and genomic DNA was ethanol precipitated and subjected to semi-quantitative PCR using primers specific for mRFP: 5′-CCC CGT AAT GCA GAA GAA GA-3′ and 5′-CTT GGC CAT GTA GGT GGT CT-3′. All genomic DNA samples were normalized with their GAPDH level using human and mouse GAPDH primers: for human, 5′-CAA CGA ATT TGG CTA CAG CA-3′ and 5′-AGG GGA GAT TCA GTG TGG TG-3′; and for mouse, 5′-ACC CAG AAG ACT GTG GAT GG-3′ and 5′-CAC ATT GGG GGT AGG AAC AC-3′. A touchdown PCR program was carried out as described [22], [24], [30]–[33]: 94°C for 2 min for 1 cycle; 92°C for 20 s, 68°C for 30 s, and 72°C for 12 cycles decreasing 1°C per cycle; and then at 92°C for 20 s, 57°C for 30 s, and 72°C for 20 s for 20–25 cycles. PCR products were resolved and visualized on 1.2% agarose gels.

### FACS analysis

Subconfluent HEK-293 or iMEF stable cells were collected at 36 h after plating, washed with PBS, and subjected to flow cytometric analysis of RFP-expressing cells using the BD LSR II Flow Cytometer and the FlowJo software. Parental HEK-293 and iMEF cells were used as blank controls. Each assay condition was done in triplicate.

### Firefly luciferase assay

For firefly luciferase reporter assay, subconfluent HEK-293 or iMEF stable cells were lyzed at 36 h after plating, and collected for measuring luciferase activity using the Luciferase Assay Kit (Progema, Madison, WI) as described [34]–[37]. Each assay condition was done in triplicate.

### Statistical analysis

Quantitative experiments were performed in triplicate and/or repeated three times. Data were expressed as mean ±SD. Statistical significances between vehicle treatment *vs*. drug-treatment were determined by one-way analysis of variance and the Student's *t* test. A value of *p*<0.05 was considered statistically significant.

## Results and Discussion

### *piggyBac* transposon vectors mediate an efficient and stable integration of transgenes in HEK-293 cells and mesenchymal stem cells iMEFs

**Figure 1:**
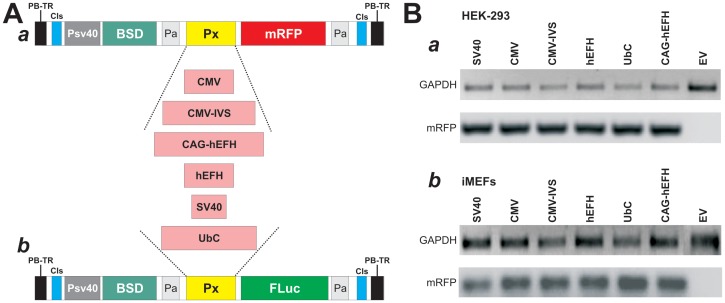
*piggyBac* transposon vectors that stably express RFP or firefly luciferase driven by different eukaryotic promoters. (**A**) Schematic representation of the piggyBac transposon vectors used in this study. The homemade *piggyBac* transposon vector pMPB was used as a base vector. Six forms of promoters were subcloned into the linker sites to drive the expression of monomeric red fluorescent protein (mRFP) (***a***) or firefly luciferase (FLuc) (***b***) as described in Methods. PB-TR, *piggyBac* terminal repeat elements; CIs, core insulators; BSD, blasticidin S selection marker; Pa, SV40 poly A signal. (**B**) Verification of the *piggyBac* vector-mediated stable integrations in HEK-293 cells and iMEFs. Stable HEK-293 (***a***) and iMEF (***b***) lines were established using the six promoter vectors and pMPB empty vector (EV) as described in Methods. Genomic DNA was isolated from these lines, and subjected to touchdown PCR analysis using the primer pair specific for mRFP or FLuc (not shown). GAPDH was used to normalize the genomic DNA level used in the PCR analysis.

In order to achieve a high level of transgene expression in mesenchymal stem cells (MSCs), we conducted a comprehensive analysis of six commonly-used constitutive promoters for their abilities to drive reporter genes RFP and luciferase in the stable lines of MSCs and the widely used somatic line HEK-293 cells. The six commonly-used promoters include three viral promoters (CMV, CMV-IVS, and SV40), one housekeeping gene promoter (UbC), and two composite promoters of viral and housekeeping gene promoters (human elongation factor α/HIV enhancer composite promoter, or hEFH, and CAG promoter linked with hEFH, or CAG-hEFH) (**Figure 1A**). We took advantage of the *piggyBac* transposon system and subcloned the six types of promoters in front of RFP (**Figure 1A, panel a**) or luciferase (**Figure 1A, panel b**) gene.

The verified vectors were co-transfected with the *piggyBac* transposase expression vector into HEK-293 and iMEF cells under the same condition. Stable cell pools for each promoter were obtained in a week. To assess the integration status of the *piggyBac* vectors, we isolated the genomic DNA from each stable cell lines and detected the presence of transgenes by PCR analysis. We found that the integration levels of the six promoter-driven RFP vectors were relatively consistent in both HEK-293 and iMEF cells (**Figure 1B**). We also performed quantitative PCR analysis and found that the copy numbers of the integrated transgene (RFP) were comparable within the same lines (data not shown). Similar results were obtained for the stable lines derived from the six promoter-driven luciferase vectors (data not shown). These results demonstrate that the *piggyBac*-based vectors achieved a similar level of integration efficiency for the tested promoters in the tested cell lines.

### CMV-based promoters drive the highest level of stable transgene expression in HEK-293 cells

**Figure 2:**
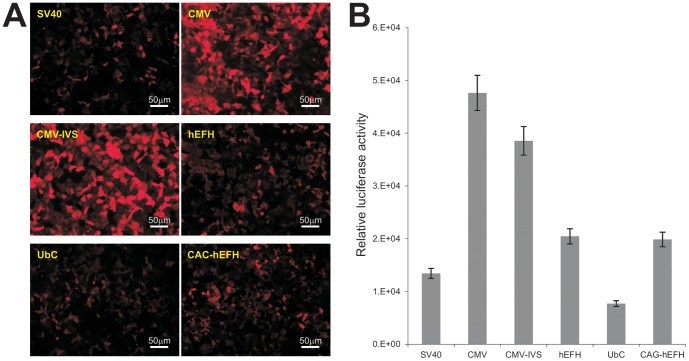
CMV-based promoters drive the highest transgene expression in somatic HEK-293 cells. (**A**) RFP expression of the stable HEK-293 cells derived from the six different promoters. Exponentially growing stable cells were seeded at subconfluency. RFP expression was recorded at 36 h after plating under the same condition for all lines. Representative results are shown. (**B**) The *piggyBac* transposon vectors expressing firefly luciferase driven by the six types of promoters were used to establish stable HEK-293 and iMEF lines. Exponentially growing stable HEK-293 cells were seeded at subconfluence and lysed at 36 h after plating for luciferase activity analysis using the Luciferase Assay System kit (Promega). Each assay conditions were done in triplicate.

The cytomegalovirus promoter CMV is one of the most commonly used and also the strongest constitutive promoters. When the RFP expression driven the six promoters in the stable 293 lines was compared, we found that the CMV and CMV-IVS promoters exhibited the highest RFP expression, while the RFP expression driven by hEFH, CAG-hEFH, and SV40 promoters was readily detectable (**Figure 2A**). The UbC promoter was shown to be weakest among the six to drive RFP expression in HEK-293 cells.

**Figure 3:**
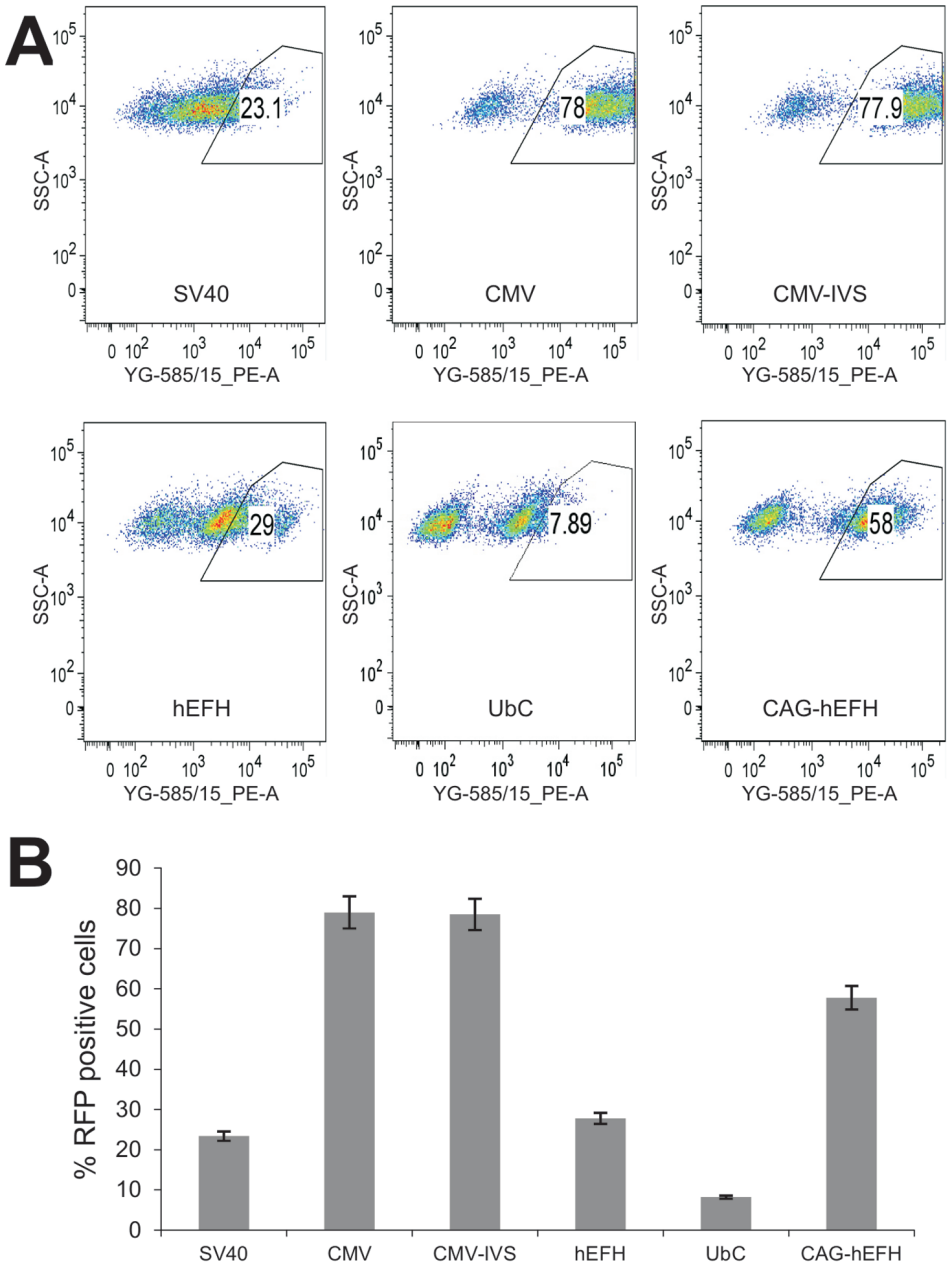
FACS analysis of RFP-positive cells in stable HEK-293 cells. Exponentially growing stable 293 cells derived from the six different promoters were seeded at subconfluence, collected at 36(**A**). Average % of RFP positive cells for the stable lines was plotted (**B**).

We further carried out two quantitative analyses of the promoter activities. First, using firefly luciferase reporter gene, we found that CMV and CMV-IVS promoters yielded the highest luciferase activities, followed by hEFH, CAG-hEFH, and SV40 promoters, whereas UbC was the weakest promoter in driving luciferase expression (**Figure 2B**). Secondly, we conducted FACS analysis to determine the percentage of RFP-positive cells in the HEK-293 stable lines (**Figure 3A**). The highest RFP-positivity was found in CMV and CMV-IVS-driven RFP stable lines, followed by CAG-hEFH promoter, to a lesser extent, hEFH and SV40 promoters, whereas UbC promoter yielded the lowest percentage of RFP-positive cells (**Figure 3B**). It's noteworthy that we further found that the CMV promoter is the strongest in several human cancer lines, including HCT116, 143B, MG63, and SW480 (data not shown). Taken together, these results indicate that the constitutive viral promoters CMV and CMV-IVS exhibit the strongest ability to drive *piggyBac* transposon-mediated stable transgene expression in HEK-293 cells.

### The hEFH composite promoter drives the highest level of stable transgene expression in mesenchymal stem cells (MSCs)

**Figure 4:**
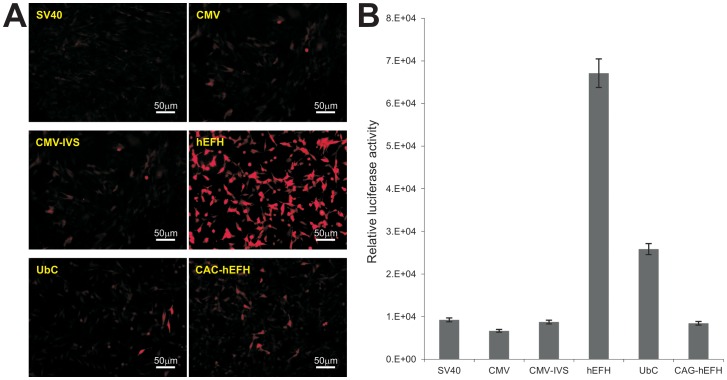
Human elongation factor α/HIV enhancer hybrid promoter (hEFH) drives the highest level of transgene expression in mesenchymal stem cells. (**A**) RFP expression of the stable iMEFs derived from the six different promoters. Exponentially growing stable cells were seeded at subconfluence. RFP expression was recorded at 36 h after plating under the same condition for all lines. Representative results are shown. (**B**) The *piggyBac* transposon vectors expressing firefly luciferase driven by the six types of promoters were used to establish stable iMEs. Exponentially growing stable iMEFs were seeded at subconfluence and lysed at 36 h after plating for luciferase activity analysis using the Luciferase Assay System kit (Promega). Each assay conditions were done in triplicate.

**Figure 5:**
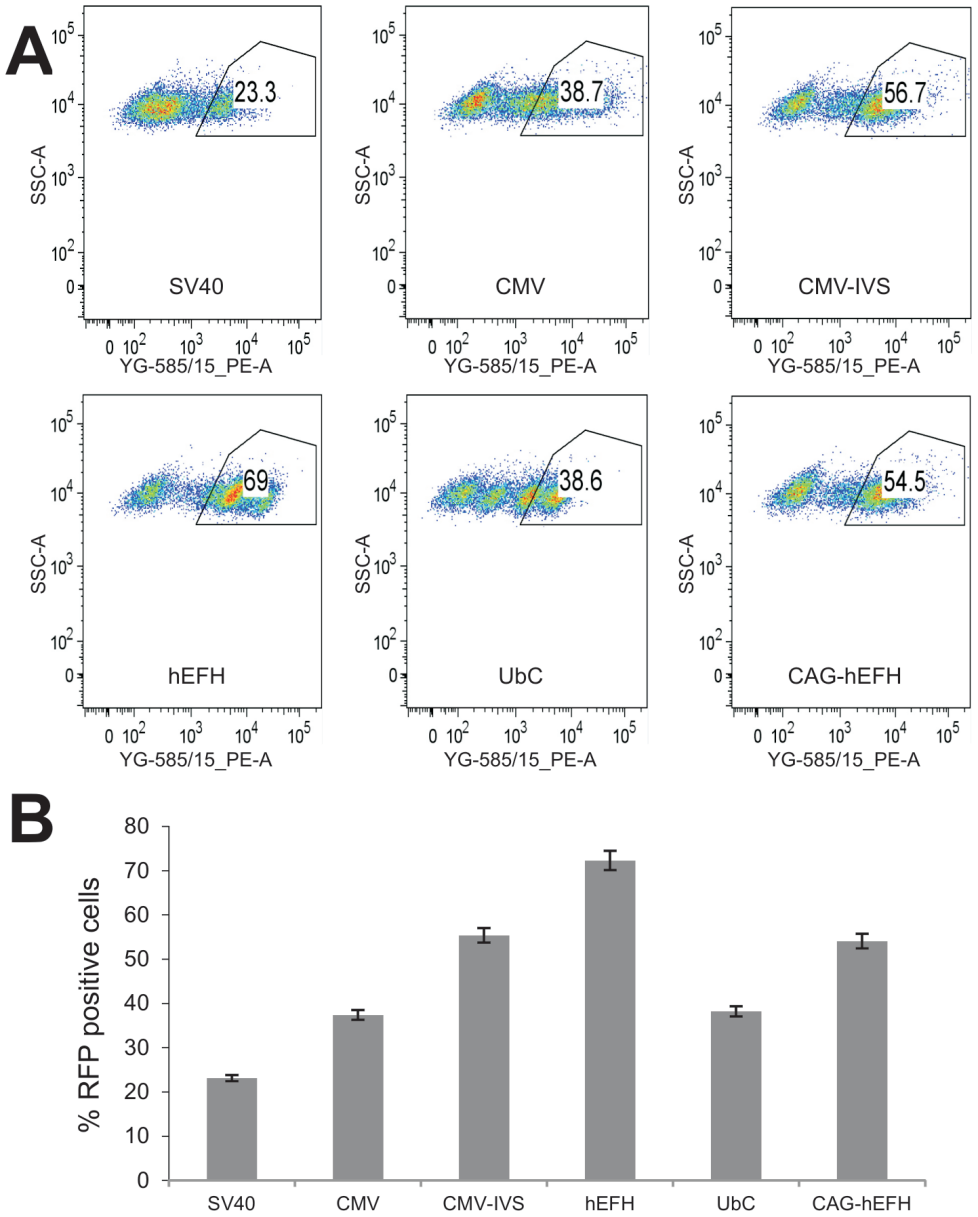
FACS analysis of RFP-positive cells in stable iMEFs. Exponentially growing stable iMEFs derived from the six different promoters were seeded at subconfluence, collected at 36(**A**). Average % of RFP positive cells for the stable lines was plotted (**B**).

It is generally believed that chromatins are mostly in repression model and thus gene expression is more tightly regulated in stem cells [13]–[17]. Thus, we compared the promoter activities in MSCs. Using our previously established and characterized MSC line iMEFs [21], we established stable lines expressing RFP or firefly luciferase driven under the control of the six promoters. Unlike in HEK-293 cells, we found that the highest RFP expression was found in the hEFH-driven RFP stable iMEFs, whereas detectable but significantly reduced RFP signal in the iMEF stable lines derived from CMV, CMV-IVS, SV40, UbC, and CAG-hEFH promoters (**Figure 4A**). Similar results were obtained from the luciferase stable lines derived from these promoters. The hEFH promoter drove the highest luciferase activity although the UbC promoter exhibited an appreciable luciferase activity (**Figure 4B**). FACS analysis demonstrated that although most stable lines exhibited between 20–50% positivity (**Figure 5A**), the hEFH promoter yielded the highest percentage of RFP-positive cells in the iMEF stable line (**Figure 5B**). Collectively, these results demonstrate that the hEFH composite promoter drives the highest transgene expression of stable iMEF lines using the *piggyBac* transposon vector.

### Transgene expression driven by CMV promoter, but not hEFH promoter, is silenced by DNA methylation and, to a lesser extent, histone deacetylation in mesenchymal stem cells

**Figure 6:**
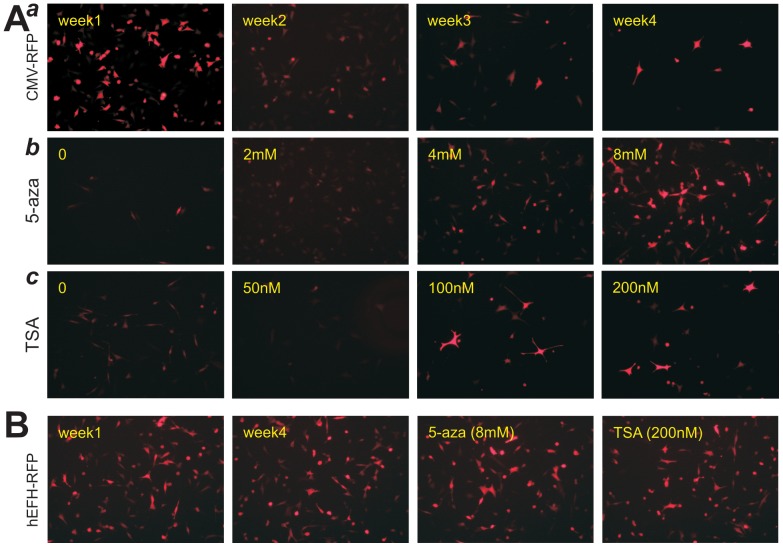
Transgene expression driven by CMV promoter, but not hEFH promoter, is silenced by DNA methylation and, to a lesser extent, histone deacetylation in mesenchymal stem cells. (**A**) Stable iMEF lines expressing RFP driven by CMV promoter were selected and cultured for up to 4 weeks (***a***), or the stable line was treated with the indicated concentrations of 5-azacytidine (***b***) or histone deacetylase (HDAC) inhibitor trichostatin A (TSA) (***c***). RFP expression was recorded at 48 h post treatment. (**B**) The hEFH-driven RFP expression was not affected in iMEFs by 5-azacytidine (8 mM) or trichostatin A (200 nM). All RFP images were recorded under the same condition. All treatment groups were done in duplicate. Representative results are shown.

We conducted further analysis of the CMV promoter-driven transgene expression in MEFs. We found that during the process of stabling stable line, CMV-driven RFP expression steadily decreased in the first 2–3 weeks after drug selection (**Figure 6A** **, panel a**). We checked if the reduced transgene expression driven by the CMV promoter in MSCs was caused by epigenetic modifications. When the long-term stable line derived from CMV-RFP was treated with the demethylation agent 5-azacytidine (5-aza), the RFP expression was restored in a dose-dependent manner (**Figure 6A** **, panel b**). When the stable line was treated with the histone deacetylase (HDac) inhibitor trichostatin A (TSA), the RFP expression was partially restored (**Figure 6A** **, panel c**). Conversely, the iMEF stable line derived from hEFH-RFP was shown to express a high level of RFP, which was not significantly affected by either 5-azacytidine or TSA (**Figure 6B**). Thus, our results strongly suggest that methylation of CMV promoter and to a lesser extent histone deacetylation may be at least in part responsible for the reduced transgene expression in MSCs, and that the hEFH hybrid promoter may be refractory to these epigenetic modifications. Therefore, the hEFH promoter can be used to drive a high level of stable transgene expression in stem cells, such as mesenchymal stem cells.

### Composite hEFH promoter may be one of the best choices for driving high levels of stable transgene expression in stem cells

In this study, we used the *piggyBac* transposon vector as the means to establish stable transgene expression. The *piggyBac* transposon system offers significant advantages over the retroviral or lentiviral system [19], [20]. First, *piggyBac* vector can deliver large cargo sizes, up to 100kb of DNA fragments, into mammalian cells. Second, unlike retroviral or lentiviral infection, *piggBac* vectors can be delivered into cells with multiple copies so it is easy to achieve high levels of transgene expression. Third, *piggyBac* exhibits non-random integration site selectivity and has a higher preference for integrations in regions surrounding AT-rich transcriptional start sites [38]. We compared the RFP expression in the stable iMEFs cells established with a *piggyBac* vs. retroviral vector and found that piggyBac vector delivers significantly stronger RFP expression (data not shown). Lastly, it is conceivable that *piggyBac* transposon can be removed from the host genome by its transposase and thus leaves no footprints using the excision-only/dominant forms of mutant *piggyBac* transposase have recently been reported [39], [40]. Thus, it is conceivable that the *piggyBac* transposon-mediated transgene expression can be reversible and footprint-free. Nonetheless, it remains to be thoroughly investigated if *piggyBac* transposon is subjected to extensive epigenetic modifications in stem cells, while it is well known that retroviral and/or lentiviral vectors are extensively modified in progenitor cells [11], [12], [41], [42].

The maintenance of stem cells and their differentiation follows defined epigenetic programs, including DNA methylation, histone modifications and small non-coding RNAs that result in gene expression, morphologic and functional changes. It was shown that human embryonic stem (ES) cells were even able to silence pluripotent promoter-driven reporter genes, such as TERT or OCT4 promoter-driven EGFP, with high efficiency [18]. Studies in human ES cells found that transgene silencing was quite marked, most notably with the pCMV promoter, while the CAGG composite promoter linked to the polyoma virus mutant enhancer PyF101 yielded stable expression clones for over 120 passages [43]. Interestingly, promoter silencing may be related to the proliferation versus differentiation status of the stem cells. It was reported that, when the transcriptional activities of three ubiquitous promoters, EF1α, phosphoglycerate kinase-1 (PGK) and CMV, were compared in mouse ES cells, in undifferentiated ES cells the EGFP expression driven by the EF1α was most stable, followed by the PGK, whereas the down-regulation of EGFP expression driven by the CMV promoter was most significant during propagation up to passage 35, although a few differentiated cells with the CMV promoter showed bright EGFP expression like that with the EF1α promoter [44]. In our studies, based on the strong promoter activities of CAG in HEK-293 and hEFH in iMEFs we engineered a hybrid promoter containing both promoters (CAG-hEFH), hoping to obtain one of the strongest promoters in stem cells. To our surprise, this hybrid promoter exhibits very limited improvement in its promoter activity. In fact, it's significantly worse than that of hEFH alone. While we do not have any satisfactory explanation for these results, it may reflect the complex transcriptional regulations of these promoters in stem cells.

Epigenetic regulation of CMV promoter was further examined in transgenic animal model. It was found that silencing of CMV promoter is dependent on the site of transgene integration, except in testis, and the nature of DNA and histone methylations strongly correlate with the expression status of the reporter [45]. Mechanistically, it was shown that silenced CMV promoter interacts in vivo with Methyl CpG binding protein 2 (MeCP2), a recruiter of histone deacetylases (HDACs) and histone (H3K9) methyl transferase [45]. We demonstrate that 5-azacytidine and to a lesser extent TSA can effectively reverse epigenetic modifications of the CMV promoter in MSCs. Our findings are supported by an early report, in which firefly luciferase activity was maximally rescued by treatment with 5-azacytidine, compared with TSA, in a dose-dependent fashion in embryonic rat cardiomyoblast cells [46].

Most eukaryotic cells abundantly express polypeptide chain elongation factor-1 alpha (EF-1α) [47]. Thus, the promoter region of human EF-1α) has been developed into a versatile expression system which has a wide host range and a high efficiency of gene expression [48]. In our study, we used the hEFH composite promoter comprising human EF-1α core promoter and the R-U5′ sequenced of the HTLV type 1 long terminal repeat. Our results have demonstrated this composite promoter exhibits a strong activity and yields long lasting expression of a transgene in MSCs. Thus, the hEFH promoter should be a preferred choice to drive a stable and high level of transgene expression in progenitor cells.

## References

[1] Prockop DJ (1997) Marrow stromal cells as stem cells for nonhematopoietic tissues. Science 276: 71–74. 9082988 10.1126/science.276.5309.71

[2] Pittenger MF, Mackay AM, Beck SC, Jaiswal RK, Douglas R, et al (1999) Multilineage potential of adult human mesenchymal stem cells. Science 284: 143–147. 10102814 10.1126/science.284.5411.143

[3] Deng ZL, Sharff KA, Tang N, Song WX, Luo J, et al (2008) Regulation of osteogenic differentiation during skeletal development. Front Biosci 13: 2001–2021. 17981687 10.2741/2819

[4] Rastegar F, Shenaq D, Huang J, Zhang W, Zhang BQ, et al (2010) Mesenchymal stem cells: Molecular characteristics and clinical applications. World J Stem Cells 2: 67–80. 21607123 10.4252/wjsc.v2.i4.67 PMC3097925

[5] Shenaq DS, Rastegar F, Petkovic D, Zhang BQ, He BC, et al (2010) Mesenchymal Progenitor Cells and Their Orthopedic Applications: Forging a Path towards Clinical Trials. Stem Cells Int 2010: 519028. 21234334 10.4061/2010/519028 PMC3017936

[6] Lamplot JD, Qin J, Nan G, Wang J, Liu X, et al (2013) BMP9 signaling in stem cell differentiation and osteogenesis. Am J Stem Cells 2: 1–21. 23671813 PMC3636726

[7] Hogan BL (1996) Bone morphogenetic proteins: multifunctional regulators of vertebrate development. Genes Dev 10: 1580–1594. 8682290 10.1101/gad.10.13.1580

[8] Shi Y, Massague J (2003) Mechanisms of TGF-beta signaling from cell membrane to the nucleus. Cell 113: 685–700. 12809600 10.1016/s0092-8674(03)00432-x

[9] Luu HH, Song WX, Luo X, Manning D, Luo J, et al (2007) Distinct roles of bone morphogenetic proteins in osteogenic differentiation of mesenchymal stem cells. J Orthop Res 25: 665–677. 17290432 10.1002/jor.20359

[10] Kim JH, Liu X, Wang J, Chen X, Zhang H, et al (2013) Wnt signaling in bone formation and its therapeutic potential for bone diseases. Ther Adv Musculoskelet Dis 5: 13–31. 23514963 10.1177/1759720X12466608 PMC3582304

[11] Ellis J, Yao S (2005) Retrovirus silencing and vector design: relevance to normal and cancer stem cells? Curr Gene Ther 5: 367–373. 16101511 10.2174/1566523054546233

[12] Hotta A, Ellis J (2008) Retroviral vector silencing during iPS cell induction: an epigenetic beacon that signals distinct pluripotent states. J Cell Biochem 105: 940–948. 18773452 10.1002/jcb.21912

[13] Fisher CL, Fisher AG (2011) Chromatin states in pluripotent, differentiated, and reprogrammed cells. Curr Opin Genet Dev 21: 140–146. 21316216 10.1016/j.gde.2011.01.015

[14] Ng HH, Surani MA (2011) The transcriptional and signalling networks of pluripotency. Nat Cell Biol 13: 490–496. 21540844 10.1038/ncb0511-490

[15] Orkin SH, Hochedlinger K (2011) Chromatin connections to pluripotency and cellular reprogramming. Cell 145: 835–850. 21663790 10.1016/j.cell.2011.05.019 PMC4858411

[16] Young RA (2011) Control of the embryonic stem cell state. Cell 144: 940–954. 21414485 10.1016/j.cell.2011.01.032 PMC3099475

[17] Beisel C, Paro R (2011) Silencing chromatin: comparing modes and mechanisms. Nat Rev Genet 12: 123–135. 21221116 10.1038/nrg2932

[18] Stewart R, Yang C, Anyfantis G, Przyborski S, Hole N, et al (2008) Silencing of the expression of pluripotent driven-reporter genes stably transfected into human pluripotent cells. Regen Med 3: 505–522. 18588473 10.2217/17460751.3.4.505

[19] Kim A, Pyykko I (2011) Size matters: versatile use of PiggyBac transposons as a genetic manipulation tool. Mol Cell Biochem 354: 301–309. 21516337 10.1007/s11010-011-0832-3

[20] Di Matteo M, Matrai J, Belay E, Firdissa T, Vandendriessche T, et al (2012) PiggyBac toolbox. Methods Mol Biol 859: 241–254. 22367876 10.1007/978-1-61779-603-6_14

[21] Huang E, Bi Y, Jiang W, Luo X, Yang K, et al (2012) Conditionally Immortalized Mouse Embryonic Fibroblasts Retain Proliferative Activity without Compromising Multipotent Differentiation Potential. PLoS One 7: e32428. 22384246 10.1371/journal.pone.0032428 PMC3285668

[22] Li M, Chen Y, Bi Y, Jiang W, Luo Q, et al (2013) Establishment and characterization of the reversibly immortalized mouse fetal heart progenitors. Int J Med Sci 10: 1035–1046. 23801891 10.7150/ijms.6639 PMC3691803

[23] Liu X, Qin J, Luo Q, Bi Y, Zhu G, et al.. (2013) Cross-talk between EGF and BMP9 signalling pathways regulates the osteogenic differentiation of mesenchymal stem cells. J Cell Mol Med. 10.1111/jcmm.12097 PMC4118175 23844832

[24] Zhang J, Weng Y, Liu X, Wang J, Zhang W, et al (2013) Endoplasmic reticulum (ER) stress inducible factor cysteine-rich with EGF-like domains 2 (Creld2) is an important mediator of BMP9-regulated osteogenic differentiation of mesenchymal stem cells. PLoS One 8: e73086. 24019898 10.1371/journal.pone.0073086 PMC3760886

[25] Kong Y, Zhang H, Chen X, Zhang W, Zhao C, et al (2013) Destabilization of Heterologous Proteins Mediated by the GSK3beta Phosphorylation Domain of the beta-Catenin Protein. Cell Physiol Biochem 32: 1187–1199. 24335169 10.1159/000354518 PMC4064945

[26] Gao Y, Huang E, Zhang H, Wang J, Wu N, et al (2013) Crosstalk between Wnt/beta-Catenin and Estrogen Receptor Signaling Synergistically Promotes Osteogenic Differentiation of Mesenchymal Progenitor Cells. PLoS One 8: e82436. 24340027 10.1371/journal.pone.0082436 PMC3855436

[27] Luo Q, Kang Q, Song WX, Luu HH, Luo X, et al (2007) Selection and validation of optimal siRNA target sites for RNAi-mediated gene silencing. Gene 395: 160–169. 17449199 10.1016/j.gene.2007.02.030

[28] Miyazaki J, Takaki S, Araki K, Tashiro F, Tominaga A, et al (1989) Expression vector system based on the chicken beta-actin promoter directs efficient production of interleukin-5. Gene 79: 269–277. 2551778 10.1016/0378-1119(89)90209-6

[29] Niwa H, Yamamura K, Miyazaki J (1991) Efficient selection for high-expression transfectants with a novel eukaryotic vector. Gene 108: 193–199. 1660837 10.1016/0378-1119(91)90434-d

[30] Sharff KA, Song WX, Luo X, Tang N, Luo J, et al (2009) Hey1 Basic Helix-Loop-Helix Protein Plays an Important Role in Mediating BMP9-induced Osteogenic Differentiation of Mesenchymal Progenitor Cells. J Biol Chem 284: 649–659. 18986983 10.1074/jbc.M806389200 PMC2610517

[31] Bi Y, Huang J, He Y, Zhu GH, Su Y, et al (2009) Wnt antagonist SFRP3 inhibits the differentiation of mouse hepatic progenitor cells. J Cell Biochem 108: 295–303. 19562671 10.1002/jcb.22254

[32] Zhu GH, Huang J, Bi Y, Su Y, Tang Y, et al. (2009) Activation of RXR and RAR signaling promotes myogenic differentiation of myoblastic C2C12 cells. Differentiation 78: 195–204. 10.1016/j.diff.2009.06.001 PMC2829657 19560855

[33] Wang Y, Hong S, Li M, Zhang J, Bi Y, et al.. (2013) Noggin resistance contributes to the potent osteogenic capability of BMP9 in mesenchymal stem cells. J Orthop Res. 10.1002/jor.22427 23861103

[34] Zhang W, Zhang H, Wang N, Zhao C, Zhang H, et al (2013) Modulation of beta-Catenin Signaling by the Inhibitors of MAP Kinase, Tyrosine Kinase, and PI3-Kinase Pathways. Int J Med Sci 10: 1888–1898. 24324366 10.7150/ijms.6019 PMC3856380

[35] Yang K, Chen J, Jiang W, Huang E, Cui J, et al (2012) Conditional Immortalization Establishes a Repertoire of Mouse Melanocyte Progenitors with Distinct Melanogenic Differentiation Potential. J Invest Dermatol 132: 2479–2483. 22592154 10.1038/jid.2012.145 PMC4083699

[36] Wang X, Cui J, Zhang BQ, Zhang H, Bi Y, et al.. (2013) Decellularized liver scaffolds effectively support the proliferation and differentiation of mouse fetal hepatic progenitors. J Biomed Mater Res A. 10.1002/jbm.a.34764 PMC4056447 23625886

[37] Shui W, Yin L, Luo J, Li R, Zhang W, et al.. (2013) Characterization of chondrocyte scaffold carriers for cell-based gene therapy in articular cartilage repair. J Biomed Mater Res A. 10.1002/jbm.a.34661 PMC4056444 23629940

[38] Wilson MH, Coates CJ, George AL Jr (2007) PiggyBac transposon-mediated gene transfer in human cells. Mol Ther 15: 139–145. 17164785 10.1038/sj.mt.6300028

[39] Li X, Burnight ER, Cooney AL, Malani N, Brady T, et al (2013) piggyBac transposase tools for genome engineering. Proc Natl Acad Sci U S A 110: E2279–2287. 23723351 10.1073/pnas.1305987110 PMC3690869

[40] Yusa K, Zhou L, Li MA, Bradley A, Craig NL (2011) A hyperactive piggyBac transposase for mammalian applications. Proc Natl Acad Sci U S A 108: 1531–1536. 21205896 10.1073/pnas.1008322108 PMC3029773

[41] Choi KH, Park JK, Kim HS, Uh KJ, Son DC, et al (2013) Epigenetic changes of lentiviral transgenes in porcine stem cells derived from embryonic origin. PLoS One 8: e72184. 23977247 10.1371/journal.pone.0072184 PMC3747048

[42] Rival-Gervier S, Lo MY, Khattak S, Pasceri P, Lorincz MC, et al (2013) Kinetics and epigenetics of retroviral silencing in mouse embryonic stem cells defined by deletion of the D4Z4 element. Mol Ther 21: 1536–1550. 23752310 10.1038/mt.2013.131 PMC3734652

[43] Liew CG, Draper JS, Walsh J, Moore H, Andrews PW (2007) Transient and stable transgene expression in human embryonic stem cells. Stem Cells 25: 1521–1528. 17379764 10.1634/stemcells.2006-0634

[44] Wang R, Liang J, Jiang H, Qin LJ, Yang HT (2008) Promoter-dependent EGFP expression during embryonic stem cell propagation and differentiation. Stem Cells Dev 17: 279–289. 18447643 10.1089/scd.2007.0084

[45] Mehta AK, Majumdar SS, Alam P, Gulati N, Brahmachari V (2009) Epigenetic regulation of cytomegalovirus major immediate-early promoter activity in transgenic mice. Gene 428: 20–24. 18976699 10.1016/j.gene.2008.09.033

[46] Krishnan M, Park JM, Cao F, Wang D, Paulmurugan R, et al (2006) Effects of epigenetic modulation on reporter gene expression: implications for stem cell imaging. Faseb J 20: 106–108. 16246867 10.1096/fj.05-4551fje PMC3625424

[47] Wakabayashi-Ito N, Nagata S (1994) Characterization of the regulatory elements in the promoter of the human elongation factor-1 alpha gene. J Biol Chem 269: 29831–29837. 7961976

[48] Kim DW, Uetsuki T, Kaziro Y, Yamaguchi N, Sugano S (1990) Use of the human elongation factor 1 alpha promoter as a versatile and efficient expression system. Gene 91: 217–223. 2210382 10.1016/0378-1119(90)90091-5

